# An Exploratory Study of Imagining Sounds and “Hearing” Music in Autism

**DOI:** 10.1007/s10803-019-04346-w

**Published:** 2019-12-20

**Authors:** Alex Bacon, C. Philip Beaman, Fang Liu

**Affiliations:** School of Psychology and Clinical Language Sciences, University of Reading, Earley Gate, Whiteknights, Reading, RG6 6AL UK

**Keywords:** Autism, Auditory imagery, Earworms, Music

## Abstract

Individuals with autism spectrum disorder (ASD) reportedly possess preserved or superior music-processing skills compared to their typically developing counterparts. We examined auditory imagery and earworms (tunes that get “stuck” in the head) in adults with ASD and controls. Both groups completed a short earworm questionnaire together with the Bucknell Auditory Imagery Scale. Results showed poorer auditory imagery in the ASD group for all types of auditory imagery. However, the ASD group did not report fewer earworms than matched controls. These data suggest a possible basis in poor auditory imagery for poor prosody in ASD, but also highlight a separability between auditory imagery and control of musical memories. The separability is present in the ASD group but not in typically developing individuals.

## Introduction

Musical ability is dependent upon a group of auditory perceptual and cognitive skills which may be affected by autism in ways that are poorly understood. The relationship between language and music is itself complex. In 1871, Darwin speculated that they might share a common evolutionary origin (Darwin 1871), and the two seem to involve distinct but overlapping mechanisms (Patel 2012). Both are reliant upon the construction of meaningful structures from a set of discrete elements (phonemes, tones) with little inherent meaning. The problem for the listener in each case is to extract meaning from acoustically variable signals where, for example, phonetic or local melodic context might modulate the intended meaning.

Early studies suggested that the processing of music may be preserved in autism even when language is impaired (Applebaum et al. 1979). For example, Heaton et al. (1999) reported no significant deficit, relative to typically developing individuals, in a sample of children with ASD in associating musical melodies to appropriate emotional expressions. These results from Heaton et al. (1999) suggest that emotional valence cues are equivalently available for ASD individuals and typically developing individuals (Molnar-Szakacs and Heaton 2012). Music is also successfully employed as a therapeutic tool amongst this population (Geretsegger et al. 2014; Janzen and Thaut 2018; Sharda et al. 2018). Forms of music therapy have been reported to aid people with severe ASD significantly improve in musical skill in different ways, such as short/long melody recall and rhythm reproduction (Boso et al. 2007). However, while some results are consistent with this idea of enhanced musical ability in autism, with reports of increased low-level pitch processing (Bonnel et al. 2003), enhanced short- and long-term pitch memory (Stanutz et al. 2014), and superior musical processing abilities in ASD (Jiang et al. 2015; Molnar-Szakacs and Heaton 2012), a few more recent studies have also found normal (Germain et al. 2019) or impaired (Sota et al. 2018) performance on pitch discrimination and melodic perception in individuals with ASD. This discrepancy may partly be explained by differences in autism severity between the tested samples: those with enhanced musical abilities tend to have language delay or impairments and those with normal musical processing are generally high functioning (Bonnel et al. 2010; Heaton et al. 2008; Jones et al. 2009; Mayer et al. 2016). For example, Chowdhury et al. (2017) and Jones et al. (2009) both found little evidence for group differences in pitch or frequency perception between ASD and control groups. However, Jones et al. (2009) reported a subgroup of ASD individuals with average intellectual ability and superior frequency discrimination skills, consistent with earlier work.

Contradictory findings have also been observed in neurophysiological and event-related potential (ERP) studies of pitch processing in ASD. A recent meta-analysis of results using these techniques suggests that infants and children (but not adults) with ASD have impaired auditory brainstem response to sound (Miron et al. 2018). There are mixed reports regarding cortical sound encoding (P1-N1-P2), with enhanced (Ferri et al. 2003), normal (Čeponienė et al. 2003), and impaired patterns (Roberts et al. 2011; Whitehouse and Bishop 2008) all reported. Discrepant results for auditory mismatch negativity (MMN) have also been reported in ASD (Schwartz et al. 2018), ranging between enhanced (Gomot et al. 2011; Lepistö et al. 2007), normal (Čeponienė et al. 2003) and impaired patterns (Jansson-Verkasalo et al. 2003). ASD individuals have also been reported as showing either enhanced (Gomot et al. 2011), normal (Whitehouse and Bishop 2008), or impaired (Čeponienė et al. 2003) orientation toward sounds (P3a) and normal/impaired language-semantic processing (N4) (McCleery et al. 2010; Pijnacker et al. 2010). They show impaired voluntary attention to sounds (P3b) (Courchesne et al. 1989) especially in terms of amplitude (Cui et al. 2017) and atypical syntactic processing (P6) (Koolen et al. 2014).

The literature on auditory imagery is extensive (for a recent review, see Hubbard 2018) but very few studies of imagery have been conducted amongst ASD populations. Those imagery studies which have been conducted have tended to examine visual imagery, perhaps because of an assumption that ASD individuals are more likely to be “visual thinkers” (Grandin 2005, 2009). Hence, there is a mixed picture of musical and auditory processing ability in ASD and an almost complete lack of knowledge about how music—and sounds more generally—are conceived by individuals with ASD.

In the current study, we make use of the Bucknell Auditory Imagery Scale (BAIS; Halpern 2015) which consists of vividness and control subscales. Both subscales ask participants to begin by imaging a sound (e.g., a saxophone solo) but whereas the vividness subscale asks participants to rate the vividness of the sound (from 1- no image present at all to 7-as vivid as the actual sound) the control subscale asks the participant how easy they find it to imagine a change (e.g., the saxophone is now accompanied by a piano).

The vividness subscale (BAIS-V) predicts vocal pitch imitation accuracy (“singing in pitch”; Greenspon et al. 2017; Pfordresher and Halpern 2013) in the general population but not pitch perception per se (Pfordresher and Halpern 2013). To the extent that auditory capabilities such as pitch perception are required to provide content for cognitive processes and to inform vivid musical memories and other auditory images, one might therefore anticipate that individuals with ASD might report vivid auditory imagery, possibly more so than a matched control group. However, Pruitt et al. (2019) suggest that covert activation of auditory images is also required for vocal pitch imitation in language learning—particularly tone languages—as well as singing and this would suggest, given the impaired prosody which often accompanies ASD as well as the frequent language delay or impairment (Mody et al. 2013), that individuals with ASD might perform poorly on such a measure. Consistent with this, the vividness scores of the BAIS correlate with gray matter volume in sensorimotor regions of the brain, specifically supplementary motor area (SMA), parietal cortex, medial superior frontal gyrus and middle frontal gyrus, where SMA and parietal systems were also reported as engaged by auditory processing (Lima et al. 2015).

The BAIS control (BAIS-C) subscale predicts performance on musical imagery related tasks (Gelding et al. 2015) and sensorimotor synchronization both in terms of absolute synchrony with a beat and anticipatory timing (predicting, rather than reacting to, beat intervals; Colley et al. 2018). The individual is asked to imagine a particular auditory experience (which might be musical, verbal, or comprised of miscellaneous environmental sounds) and either to self-rate how vivid they find the experience (vividness subscale) or how easy they find it to control the experience, perhaps by transforming the imagined sound into a related but different sound. One possibility is that ASD participants might score equivalently, or better than, a control group on the BAIS-C given the report of preserved auditory-motor rhythm synchronization in children with ASD (Tryfon et al. 2017).

Additionally, factor analysis of the two subscales has identified items within each subscale which load upon three factors corresponding approximately to images of environmental sounds, music, and voice. It may be that imagery is dissociable for these factors such that ASD individuals might score equivalently, or at higher levels than controls, on musical imagery even if their imagery scores are lower for other factors such as voice/verbal stimuli. Both subscales of the BAIS also correlate equivalently with the vividness of visual imagery questionnaire-modified (VVIQ-M; McKelvie 1995). As noted previously, previous studies on mental imagery in ASD have focussed on the visual. A prototypical study is that of Scott and Baron-Cohen (1996) which found that children with ASD had particular problems with imagining “unreal” or impossible things. If this is a general principle for all forms of mental imagery, then we might also expect to find the ASD population only showing difficulties in auditory imagery where the imagined sounds are “unreal” or impossible. Since none of the BAIS items are “unreal” or impossible in the sense intended by Scott and Baron-Cohen (1996), there is no reason, from this perspective, to predict any difference between the self-reported auditory imagery of ASD and control groups.

Finally, the control subscale of the BAIS positively correlates with the movement factor of Floridou et al. (2015) involuntary musical imagery scale and both vividness and control subscales of the BAIS correlated with the number of earworms, or involuntarily-experienced tunes, induced experimentally in a study reported by Beaman (2018). Earworms or, colloquially, songs stuck in the head are a common experience in the general population. Liikkanen (2012) found that 33% of a large internet sample reported experiencing earworms daily, with 90% experiencing the phenomenon at least once a week. People who listen to more music throughout the day have been reported to have less frequent earworms, but of similar durations to others (Williamson and Jilka 2014). Strong positive correlations have been found between practicing a musical instrument and earworm frequency (Liikkanen 2012), suggesting that expertise and/or training can have an effect on the appearance of earworms. Floridou et al. (2012) did not replicate these findings, but Beaman and Williams (2010) found that the self-reported importance assigned by individuals positively correlated with the appearance of earworms. Thus, it appears that engaging in music in some way is associated with experiencing earworms. Ockelford (2015) notes that, sensory reactivity issues notwithstanding, many children with autism seek out musical experiences. Ockelford (2015) also suggests that the mental imagery particularly of those ASD children who possess superior pitch perception (in particular, those with absolute pitch) might be more vivid if it allows for direct, rather than indirect, access to memories of the original percepts. The logic of this is somewhat against what is known about the reconstructive nature of memory (Schacter et al. 1998; Surprenant and Neath 2003). Ockelford notes that, based upon (albeit informal) clinical observational evidence of children with ASD repeatedly humming, singing or whistling snippets of tunes, “earworms are a relatively common feature among this population” (Ockelford 2015, p. 133). Thus, there is at least *prima facie* reason to believe there may be more earworms amongst an ASD group as a function of more vivid auditory imagery, reflected in higher scores on BAIS.

In summary, a mixed picture of auditory and musical processing in ASD has emerged from behavioral and psychophysiological studies. Reflecting this mixed evidence, there are multiple theoretical perspectives from which a number of hypotheses can be derived regarding mental control, and subjective impressions of musical and other auditory images in ASD but no data have yet been presented which might test such hypotheses. The current study therefore takes an exploratory approach to determine what, if any, differences can be discerned between auditory imagery in an ASD and appropriately matched control group. The question addressed here is whether, given the mixed ERP and behavioral data on low-level auditory processing and musical capabilities in ASD, the experience of imagining sounds—and particularly music—is discernibly different in ASD individuals from that reported by a control group, and in what ways. The current study examines the cognitive and experiential aspects of auditory (including musical) processing and control, an area which has been hitherto neglected in studies of musicality and ASD.

## Method

### Participants

The study was approved by the University of Reading ethics committee and performed in accordance with the ethical standards as laid down in the 1964 Declaration of Helsinki and its later amendments. Participants were recruited through a variety of ways, such as word-of-mouth, contacting local organisations associated with autism, and online advertising.

The sample consisted of 34 participants: 17 ASD participants (10 female, 7 male), aged 16–56, with an average of 36.6 years old matched with 17 controls on gender. All ASD participants had an official clinical diagnosis, which is also confirmed by their AQ scores. Bayesian *t*-tests confirmed that the two groups did not differ in age, self-reported years of musical training, non-verbal IQ as measured by Raven’s progressive matrices, or receptive vocabulary as measured by the fourth edition of the Receptive One Word Picture Vocabulary Test (ROWPVT-4, see Table 1). An exclusion criterion for the control group was an AQ score over 32, consistent with the procedure of Baron-Cohen et al. (2001). An exclusion criterion for ASD participants was an AQ score of less than 32. Nevertheless, no participants were excluded on the basis of either of these two criteria in this study. Confirmatory Bayesian *t*-tests provide strong evidence that, in addition to the AQ, the two groups differed on Empathy Quotient (EQ), (*t* (32) = 5.1, *p* < .001, Cohen’s *d* = 1.75; BF_10_ = 1115.205, median = − 1.59, 95% CI − 2.42, − .77) and Systemizing Quotient (SQ) (*t*(32) = 4.7, *p* < .001, Cohen’s *d* = 1.61, BF_10_ = 400.729, median = 1.46, 95% CI .66, 2.25).

**Table 1:** Mean and standard deviations for ASD and typically developing control groups on matched demographic variables and AQ

Group	Age	Musical training	Raven’s	ROWPVT-4	AQ
ASD	36.59 (13.1)	4.82 (7.64)	53.62 (3.5)	107.19 (14.3)	41.35 (5.17)
Control	36.35 (12.4)	5.74 (6.98)	52.82 (3.97)	107.24 (16.5)	14.65 (7.03)
Comparison statistics: Bayesian					
BF_01_	3.04	2.89	2.6	3.01	1.45 × 10^−11^
Median	.10	.09	.16	.001	4.17
95% CI	− .56, .60	− .69, .48	− .43, .78	− .58, .60	2.91, 5.44
Comparison statistics: frequentist					
*t*	.05	.36	.62	.001	12.62
*p*	.96	.72	.54	.99	<.001
Cohen’s *d*	.018	.125	.21	.003	4.329

Frequentist comparisons are given by 2-tailed independent *t*-test results. Bayes factors from a default prior Bayesian *t*-test are expressed in terms of the Bayes factor in favour of the null hypothesis of no difference (BF_01_). The delta effect size is given by the median of the *a posterior* distribution and 95% credible intervals

The full data-set, including all participants, are available via the Open Science Framework at https://osf.io/q7k9n/. Three ASD participants answered all questions with only 1 (one participant) or 7 (two participants), and these participants (and their matched controls) were excluded from subsequent analyses on the presumption that they had failed to understand the task. Importantly, including these individuals does not affect the results except on the one occasion noted in the text.

## Materials

The materials used were two online questionnaires, which were provided through the JISC (formerly BOS) survey platform. First, the Bucknell Auditory Imagery Scale (BAIS) was given to participants online. The second questionnaire used was an earworms questionnaire, initially used by Beaman and Williams (2013) and shown to correlate with schizotypy and mental suppression in a non-clinical sample. Questions 1 and 5 were on a numerical scale, whereas questions 2, 3, 4, 6 and 7 were on an ordinal scale, ranging from A–C or A–E, which were converted into increasing numerical values depending on participant responses e.g. A = 1, B = 2, etc.

### Procedure

Links to both questionnaires were sent by email, for participants to complete in their own time. The AQ was sent first, then the BAIS and finally the earworms questionnaire. Time pressure was removed for participants as there was no time limit given and no supervision was given to ensure that they necessarily completed the questionnaires in this order. As part of a general recruitment procedure all individuals also subsequently completed a number of other tests, as indicated in Table 1, as a means of matching pairs on appropriate measures for this and other studies.

Bayesian analyses were run on JASP software using default priors because there were no strong a priori commitments to any particular effect size (Rouder et al. 2009; van Doorn et al. 2019). Bayesian test results are reported throughout because some of the theoretical perspectives reviewed give rise to predictions of no difference between the two groups. Unlike frequentist hypothesis tests, Bayes factors allow one to report evidence in favour of the null hypothesis, rather than simply failing to reject the null. Bayes factors give continuous measures of the likelihood of one hypothesis over another. The continuous nature of the evidence they provide means it would be inappropriate to provide cut-off values analogous to p-values for significance testing but for purposes of interpretation. Jeffreys (1939) and others have suggested ranges of values which they consider equivalent to different “strengths” of evidence, where a BF value of about 3 is the point at which evidence either for or against an hypothesis should begin to be taken seriously.

## Results

**Fig. 1:**
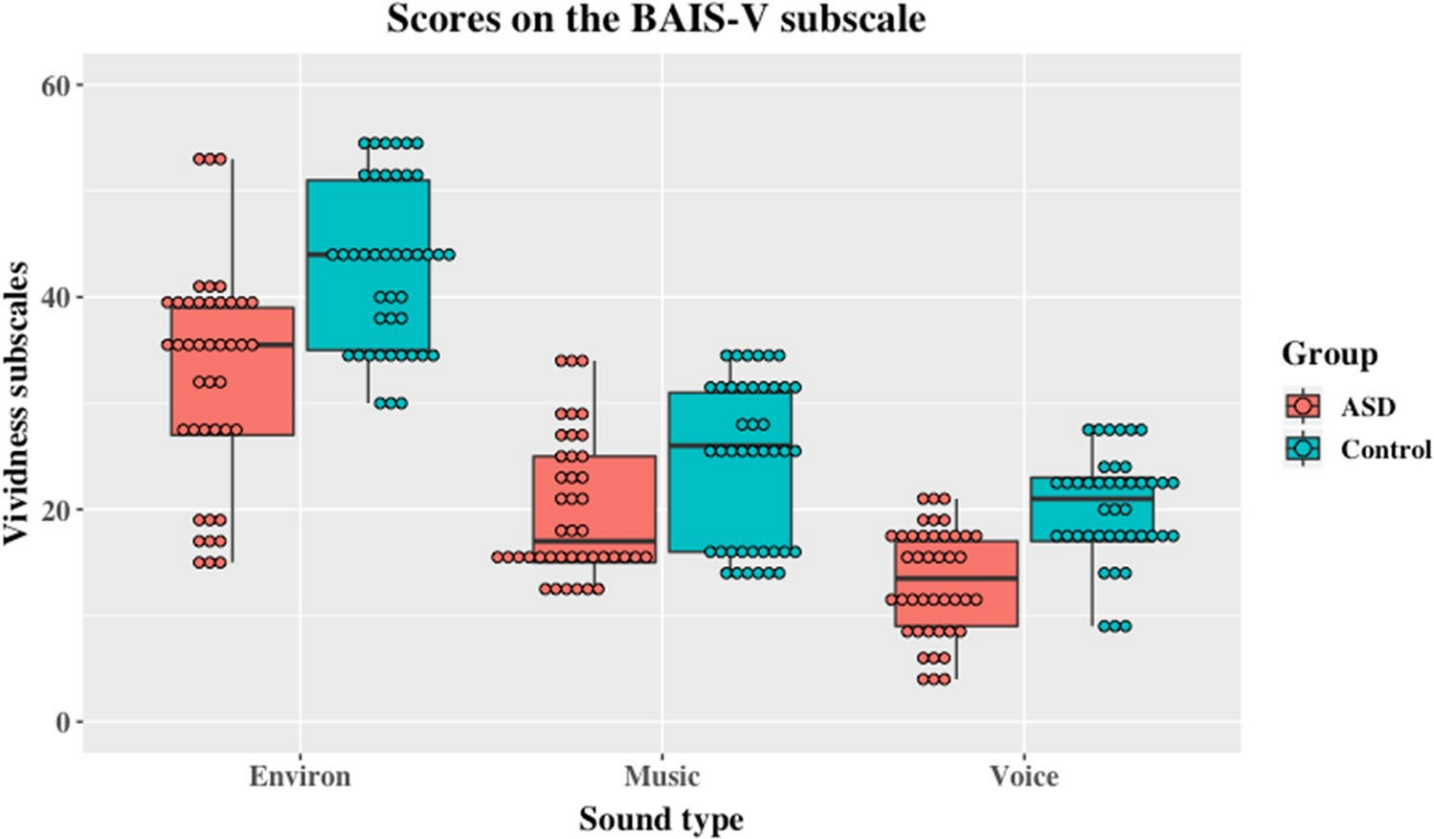
Scores on the BAIS-V subscale for ASD and control (typically developing) groups according to sound type

The three factors identified by factor analysis (Halpern 2015) as contributing to the BAIS-V and BAIS-C subscales, and corresponding approximately to questions about environmental sounds, music and voice were entered as three levels of a repeated measures vividness type factor of a mixed Bayesian analysis of variance (ANOVA) with group (ASD or control) as the between participants factor. ANOVA revealed a main effect of group on Vividness scores of the BAIS (*F*(1,26) = 8.13, *p* = .008, eta squared = .238), a main effect of type of vividness (*F*(2, 52) = 207.04, *p* < .001, partial eta squared = .89), and an interaction between group and vividness type (*F*(2, 52) = 3.31, *p* = .04, partial eta squared = .11), as shown in Fig. 1. Inclusion of the individuals believed not to have followed instructions results in a *p* value of .052 for the interaction, but the results are otherwise unaffected. Bayesian model comparisons lead to similar conclusions: Using JASP to calculate BF_inclusion_ values to indicate the extent to which Bayes the data support inclusion of the factor of interest, a Bayesian model with an effect of group was revealed to be more likely than the model of no group differences (BF_10_ = 9.106). A model with an effect of soundtype was also more likely than the null model of no difference in soundtype (BF_10_ > 1000,000). Finally, a model in which these factors interact was more likely than a model with no interaction (BF_10_ = 4.95).^1^

Follow-up analyses (2-tailed) using Bayesian *t*-tests and a Holm’s sequential Bonferroni correction (Holm 1979) for the frequentist results indicates that the differences between groups were reliable for voice and environmental sounds but not for music. For voice (*t*(26) = 3.55, *p* = .001, Cohen’s *d* = 1.34, BF_10_ = 22.37, median = − 1.1.35, 95% CI − 1.976, − .328) for environmental sounds (*t*(26) = 2.86, *p* = .008, Cohen’s *d* = 1.08, BF_10_ = 5.93, median = − .878, 95% CI − 1.693, − .137) and for music (*t*(26) = 1.69, *p* = .104, Cohen’s *d* = .64, BF_10_ = .997, median = − .48, 95% CI − 1.204, .178). Thus, the evidence favours the hypothesis that ASD individuals differ from controls in vividness of auditory imagery in two cases (voice and environmental sound), in the third case (music) the observed effect size was smaller and the results inconclusive.

**Fig. 2:**
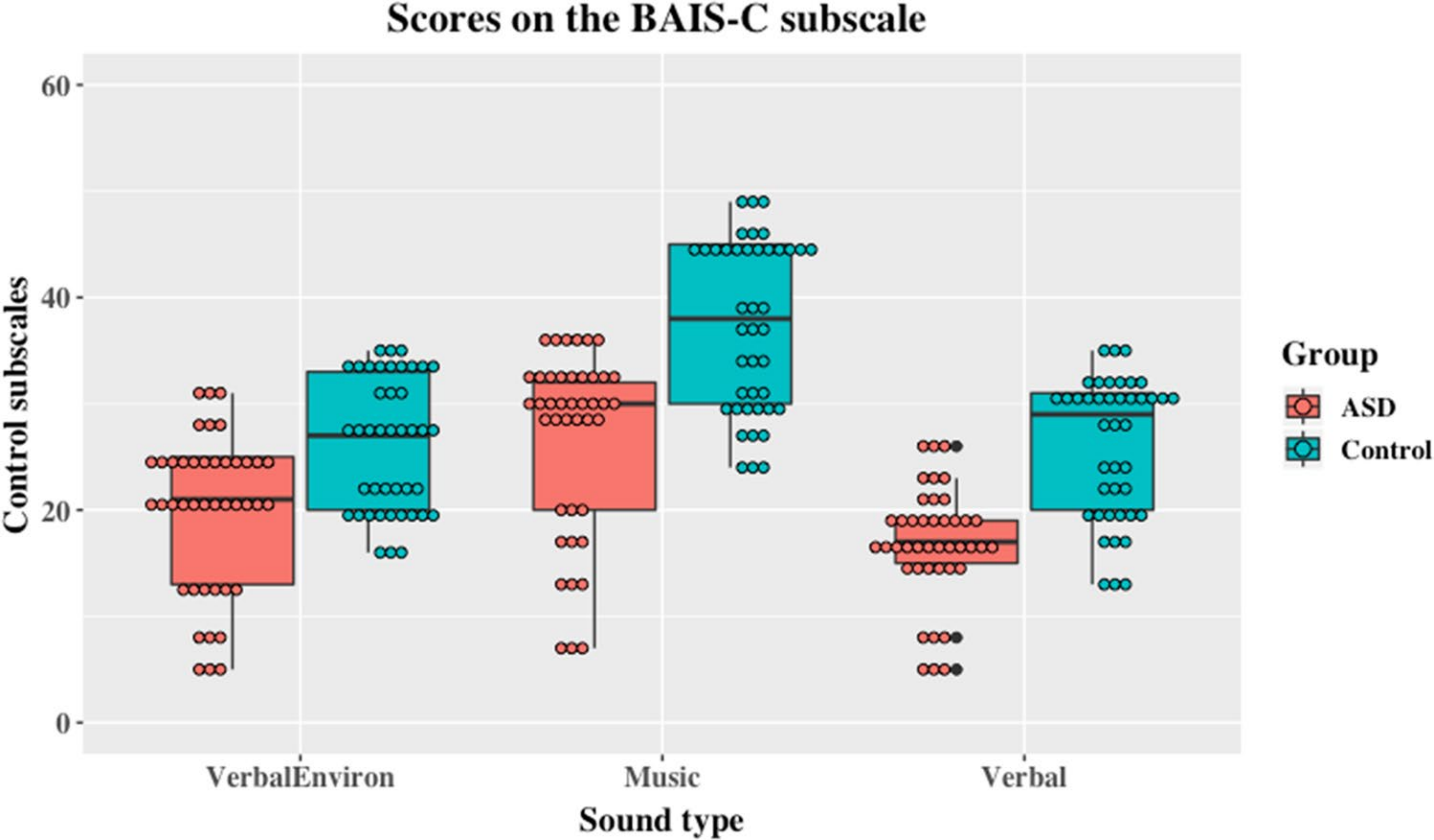
Scores on the BAIS-C subscale for ASD and control (typically developing) groups according to sound type

A similar pattern was apparent with Control scores of the BAIS where once again there was strong evidence for an effect of group (*F*(1,26) = 11.16, *p* = .003, eta squared = .3), a main effect of type of control (*F*(2, 52) = 4.74, *p* < .001, partial eta squared = .82) and a group by control type interaction (*F*(2, 52) = 118.3, *p* = .013, partial eta squared = .15), as shown in Fig. 2. Again, Bayesian model comparisons produced similar results. BF_inclusion_ values revealed that a model with an effect of group was more likely than the null model, (BF_10_ = 34.34), one with an effect of soundtype was also more likely than the null model of no effect (BF_10_ = 6.005 × 10^15)^, and a model in which both effects interact was more likely than a model with no interaction (BF_10_ = 11.58).

For BAIS-C, the three factors contributing to control scores were identifiable as arising from questions concerning verbal/environmental sounds, music, and verbal sounds (Halpern 2015). Follow-up analyses (2-tailed) using Bayesian *t*-tests and a Holm’s sequential Bonferroni correction for the frequentist results indicates that in all cases there are significant differences between the groups in verbal sounds, music, and verbal/environmental sounds (*t*(26) = 3.99, *p* < .001, Cohen’s *d* = 1.51, and *t*(26) = 3.34, *p* < .001, *p* = .003, Cohen’s *d* = 1.26, *t*(26) = 2.36, *p* = .026, Cohen’s *d* = .89, respectively) and in all cases a difference between groups is more likely than a null difference (verbal sounds: BF_10_ = 55.63, median = − 1.303, 95% CI − 2.189, − .438; music: BF_10_ = 14.51, median = − 1.051, 95% CI − 1.882, − .261; and verbal/environmental sounds: BF_10_ = 2.54, median = − .7, 95% CI − .1468, − .005). Note however that the evidence in support of the alternate hypothesis is much weaker in the case of verbal/environmental sounds than in the other instances and also that the frequentist *t* test would not have survived a stricter Bonferroni correction.

**Fig. 3:**
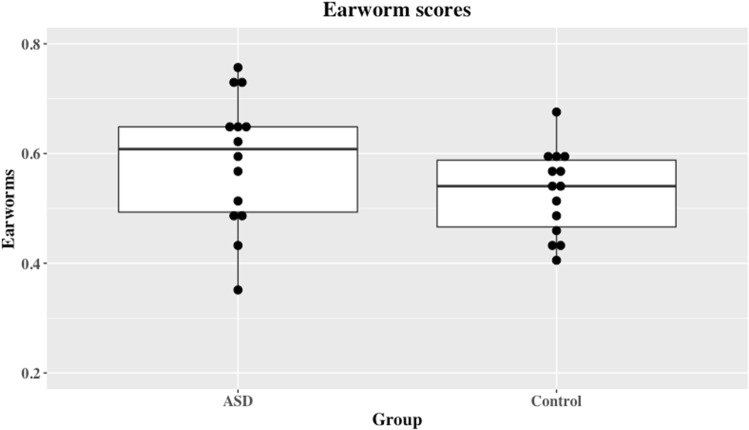
Earworm scores for ASD and control groups

Given that BAIS scores were lower amongst the ASD group than in controls, the positive correlation between BAIS scores and earworms in typically developing individuals reported by Beaman (2018) would suggest that there should be *fewer* earworms in the ASD group relative to the control group but this hypothesis is decisively rejected by a 1-tailed Bayesian *t*-test (*t* = 1.52, *df* = 26, *p* = .94, Cohen’s *d* = .57, BF_01_ = 5.997, median = − .117, 95% CI − .498, − .004). Figure 3 shows these data. Numerically there were more earworms amongst the ASD group in line with Ockelford’s (2015) speculation but the evidence in favour of such a hypothesis remains at an anecdotal level (BF_10_ = 1.48, median = .462, 95% CI .037, 1.17 when including only the individuals who successfully completed the BAIS scale; BF_10_ = 2.67, median = .551, 95% CI .06, 1.239 when all individual are included).

## Discussion

As far as we are aware, these results represent the first attempt to examine the phenomenology of auditory imagery in an ASD population and directly compare this to a matched control group. The results of this study show that both self-reported vividness and control of auditory imagery are reduced in our ASD group relative to the typically developing control group. Contrary to predictions, however, this did not result in fewer earworms and it remains possible that Ockelford’s (2015) speculation that earworms are *more* prevalent in an ASD population—the current data-set is insufficient to settle this point. It is clear, however, that any hypothesis about earworms predicated upon superior auditory imagery is not supported. It was also plausible that either or both of the vividness and control subscales would show differential effects of ASD upon the *types* of auditory imagery such that, for example, musical imagery might be preserved or even enhanced whereas vocal or verbal imagery might not be. Analyses of variance showed interaction effects, but Figs. 1 and 2 also show that even if the difference between the groups varies between types of imagery the overall trend is for ASD individuals to self-report less vivid imagery, and less control over their auditory imagery. This trend remains the same even though the difference between the groups varies between types of imagery. These data show that deficiencies in auditory imagery are not limited to impossible or fantastic situations, as has been suggested for visual imagery (Scott and Baron-Cohen 1996) and are present for both the sense of the vividness of an auditory imagery and the sense of control over that image. However, differences between the groups in how they report to different types of imagery (e.g., verbal vs musical) may reflect inherent limitations in the auditory imagery scale employed, and the relative loadings of the components.

Significantly, the Bucknell Auditory Imagery Scale was previously validated in a nonclinical population and shown to possess some interesting properties with respect to that population. Most notably, BAIS-V not only predicted individual differences in gray matter density in auditory and sensory-motor regions of the brain regions (Lima et al. 2015) but also individuals’ ability to vocally reproduce a target pitch (Pfordresher and Halpern 2013). This suggests a possible basis for poor speech production and lack of prosody in speech and language development in poor auditory imagery. If individuals rely upon auditory imagery to produce appropriately-pitched speech (whether sung or spoken), then poorer, or less vivid, auditory imagery would hinder the process of planning speech-acts as the instructions to be passed forward to the articulators would lack clarity with respect to the stress and prosody required. This difficulty with pitch production is independent of the perception of pitch in typically developing individuals (Pfordresher and Halpern 2013) and seemingly also in ASD given past reports of perfect pitch in ASD individuals (Heaton et al. 1998, 2008; Jones et al. 2009; Mottron et al. 1999).

The interpretation outlined above raises the question of why, if auditory imagery is deficient the ASD group examined here did not also show fewer earworms. Indeed, the data are roughly equally consistent with the ASD participants being equivalent to the matched controls or showing more earworms as speculated by Ockelford (2015). The most parsimonious description of these results is that auditory imagery is functionally separable from musical memories (in the form of earworms) in ASD individuals in a way it is not in typically-developing individuals.

### Strengths and Limitations of the Study

A clear limitation of the current study is the relatively small sample size, which requires us to be cautious not to over-interpret these results until such time they may be replicated with a larger sample and, perhaps, a subgroup analyses of ASD individuals. Notably, the sample size was too small for meaningful correlation coefficients to be calculated so that, for example, the correlations between BAIS scores and self-reports of earworms previously observed (Beaman 2018) were not examined for either group as the power of such a test would be low and the correlation coefficient itself unstable (Schönbrodt and Perugini 2013). The data were also insufficient to determine whether the difference in the number of earworms reported between the two groups was reliable. Ockelford’s (2015) suggestion that earworms are common in an ASD population thus remains open to future investigation, although the current data provide weak evidence consistent with this idea and the basis for an estimated effect size for future work.

Importantly, the groups were matched on a number of cognitive and demographic variables; including verbal and non-verbal ability, and age and musical training. It is not clear what results might be obtained with a less cognitively capable ASD group, but the data are clear in indicating that the differences in auditory imagery reported here cannot be explained in terms of poorer overall cognitive capability or even just a simple failure to understand the task. This is important because the literature on musical ability in ASD is mixed, with some suggestions that preserved or superior musical capability is the provenance of a subgroup of ASD individuals who show little or no cognitive impairment (Bonnel et al. 2010; Chowdhury et al. 2017; Heaton et al. 2008; Jones et al. 2009; Mayer et al. 2016). By reporting Bayes factors for the null hypothesis of no difference in the demographic and other matched variables we were able to quantify how much more likely any numerical differences were on the matching variables under the null hypothesis we wish to assert than under a (default) alternate hypothesis. The evidence in favour of the null hypothesis is not particularly substantial using Jeffreys’ (1939) Bayes factor categorisation scheme, but Bayes factors are continuous measures of the strength of evidence. Although Jeffreys’ scheme is a useful general guide, it would be misleading to reduce them to categorical decision rules in the manner of hypothesis tests. Simply providing Bayes factors as a guide goes beyond the usual practice, even in some of our own work, of concluding in favour of the null following a statistical test on (hopefully) matched participant or stimulus characteristics (e.g., Scott et al. 2009).

There are also strengths and weaknesses associated with the auditory imagery scales employed within the current study. The vividness of a mental image, being the property of introspection, is intrinsically subjective and can only be directly assessed by self-evaluation. The advantages and disadvantages attendant to a questionnaire design are discussed by Hubbard (2018). Strengths of the BAIS questionnaire identified by Hubbard (2018) include its reliability and construct validity. Converging evidence from correlations between BAIS and both behavioral and neural measures (Colley et al. 2018; Gelding et al. 2015; Greenspon et al. 2017; Halpern 2015; Lima et al. 2015; Pfordresher and Halpern 2013) further indicates that factors associated with or contingent upon auditory imagery, which have consequences for neural processing and behavioral responses, are represented within the questionnaire. Weaknesses of the questionnaire include the particular stimuli that participants are asked to imagine and which, inevitably, reflect only a subset of the possible auditory events any individual might experience. However, the success of the BAIS in enabling investigators to identify statistically reliable associations between auditory imagery and both neural characteristics (e.g. gray matter volume) and behavioral outcomes (e.g. poor pitch singing) is evidence that individuals are interpreting the questionnaire in an appropriate and productive manner. Such responses to the questionnaire are sufficient to distinguish between individuals and—as here—groups.

## Conclusions

Auditory imagery scores are shown to be lower in an ASD group than in a matched control group in terms of both the self-rated vividness and mental control experienced over imagery. This contrasts with the case of visual imagery, in which poorer mental imagery in ASD is only associated with “unreal” or impossible objects. Contrary to predictions, this poorer auditory imagery was not associated with fewer earworms amongst individuals with ASD. There was limited evidence that individuals with ASD might in fact experience more earworms than the control group, consistent with clinical observations by Ockelford (2015).

## Footnotes

1 Bayes Factors also supported an interaction effect when individuals believed not have followed instructions were included, BF_10_ = 3.41.

## References

[CR1] Applebaum E, Egel AL, Koegel RL, Imhoff B (1979). Measuring musical abilities of autistic children. Journal of Autism and Developmental Disorders.

[CR2] Baron-Cohen S, Wheelwright S, Skinner R, Martin J, Clubley E (2001). The autism-spectrum quotient (AQ): Evidence from Asperger syndrome/high-functioning autism, males and females, scientists and mathematicians. Journal of Autism and Developmental Disorders.

[CR3] Beaman CP (2018). The literary and recent scientific history of the earworm: A review and theoretical framework. Auditory Perception & Cognition.

[CR4] Beaman CP, Williams TI (2010). Earworms (“stuck song syndrome”): Towards a natural history of intrusive thoughts. British Journal of Psychology.

[CR5] Beaman CP, Williams TI (2013). Individual differences in mental control predict involuntary musical imagery. Musicae Scientae.

[CR6] Bonnel, A., McAdams, S., Smith, B., Berthiaume, C., Bertone, A., Ciocca, V., … & Mottron, L. (2010). Enhanced pure-tone pitch discrimination among persons with autism but not Asperger syndrome. *Neuropsychologia*, *48*, 2465–2475 10.1016/j.neuropsychologia.2010.04.020. 10.1016/j.neuropsychologia.2010.04.020 20433857

[CR7] Bonnel A, Mottron L, Peretz I, Trudel M, Gallun E, Bonnel A-M (2003). Enhanced pitch sensitivity in individuals with autism: A signal detection analysis. Journal of Cognitive Neuroscience.

[CR8] Boso M, Emanuele E, Minazzi V, Abbamonte M, Politi P (2007). Effect of long-term interactive music therapy on behaviour profile and musical skills in young adults with severe autism. Journal of Alternative and Complementary Medicine.

[CR9] Čeponienė E, Lepistö A, Shestakova R, Vanhala P, Alku R, Näätänen R, Yaguchi K (2003). Speech-sound-selective auditory impairment in children with autism: They can perceive but do not attend. Proceedings of the National academy of Sciences of the United States of America.

[CR10] Chowdhury, R, Sharda, M., Foster, N. E. V., Germain, E, Tryfon, A, Doyle-Thomas, K … & Hyde, K. L. (2017). Auditory pitch perception in autism spectrum disorder is associated with nonverbal abilities. *Perception, 46*, 1298–1320. 10.1177/0301006617718715 10.1177/0301006617718715 28683588

[CR11] Colley ID, Keller PE, Halpern AR (2018). Working memory and auditory imagery predict sensorimotor synchronization with expressively timed music. Quarterly Journal of Experimental Psychology.

[CR12] Courchesne E, Lincoln AJ, Yeung-Courchesne R, Elmasian R, Grillon C (1989). Pathophysiologic findings in nonretarded autism and receptive developmental language disorder. Journal of Autism and Developmental Disorders.

[CR13] Cui T, Wang PP, Liu S, Zhang X (2017). P300 amplitude and latency in autism spectrum disorder: A meta-analysis. European Child and Adolescent Psychiatry.

[CR14] Darwin C (1971). The descent of man and selection in relation to sex.

[CR15] Ferri R, Elia M, Agarwal N, Lanuzza B, Musumeci SA, Pennisi G (2003). The mismatch negativity and the P3a components of the auditory event-related potentials in autistic low-functioning subjects. Clinical Neurophysiology.

[CR101] Floridou, G. A., Williamson, V. J., & Müllensiefen, D. (2012). Contracting earworms: The role of personality and musicality. In E. Cambouropoulos, C. Tsougras, P. Mavromatis, & K. Pastidais (Eds.),* Proceedings of the 12th international conference on music perception and cognition and the 8th triennial conference of the European society for the cognitive sciences of music, ICMPC*, Thessaloniki, Greece pp. 302–309.

[CR16] Floridou G, Williamson VJ, Stewart L, Müllensiefen D (2015). The involuntary musical imagery scale (IMIS). Psychomusicology.

[CR17] Gelding RW, Thompson WF, Johnson BW (2015). The pitch imagery arrow task: Effects of musical training, vividness, and metal control. PLoS ONE.

[CR18] Geretsegger M, Elefant C, Mössler K, Gold C (2014). Music therapy for people with autism spectrum disorder. The Cochrane Library.

[CR19] Germain, E., Foster, N. E. V., Sharda, M., Chowdhury, R., Tryfon, A., Doyle-Thomas, K. A. R., … & Hyde, K. L. (2019). Pitch direction ability predicts melodic perception in autism. *Child Neuropsychology*, *25*, 445–465. 10.1080/09297049.2018.1488954. 10.1080/09297049.2018.1488954 29950145

[CR20] Gomot M, Blanc R, Clery H, Roux S, Barthelemy C, Bruneau N (2011). Candidate electrophysiological endophenotypes of hyper-reactivity to change in autism. Journal of Autism and Developmental Disorders.

[CR21] Grandin T (2005). Thinking in pictures.

[CR22] Grandin T (2009). How does visual thinking work in the mind of a person with autism? A personal account. Philosophical Transactions of the Royal Society, B.

[CR23] Greenspon EB, Pfordresher PQ, Halpern AR (2017). Pitch imitation ability in mental transformations of melodies. Music Perception.

[CR24] Halpern AR (2015). Differences in auditory imagery self-report predict behavioral and neural outcomes. Psychomusicology.

[CR25] Heaton P, Hermelin B, Pring L (1998). Autism and pitch processing: A precursor for savant musical ability?. Music Perception.

[CR26] Heaton P, Hermelin B, Pring L (1999). Can children with autism spectrum disorders perceive affect in music? An experimental investigation. Psychological Medicine.

[CR27] Heaton P, Williams K, Cummins O, Happé F (2008). Autism and pitch processing splinter skills: A group and sub-group analysis. Autism.

[CR28] Holm S (1979). A simple sequentially rejective multiple test procedure. Scandinavian Journal of Statistics.

[CR29] Hubbard TL (2018). Some methodological and conceptual considerations in studies of auditory imagery. Auditory Perception & Cognition.

[CR30] Jansson-Verkasalo, E., Ceponiene, R., Kielinen, M., Suominen, K., Jäntti, V., Linna, S. L., … & Näätänen, R. (2003). Deficient auditory processing in children with Asperger Syndrome, as indexed by event-related potentials. *Neuroscience Letters*, *338*, 197–200. 10.1016/s0304-3940(02)01405-2. 10.1016/s0304-3940(02)01405-2 12581830

[CR31] Janzen TB, Thaut MH (2018). Rethinking the role of music in the neurodevelopment of autism spectrum disorder. Music & Science.

[CR32] Jeffreys H (1939). Theory of probability.

[CR33] Jiang J, Liu F, Wan X, Jiang C (2015). Perception of melodic contour and intonation in autism spectrum disorder: Evidence from Mandarin speakers. Journal of Autism and Developmental Disorders.

[CR34] Jones, C. R. G., Happé, F., Baird, G., Simonoff, E., Marsden, A. J. S., Tregay, J ….. & Charman, T. (2009). Auditory discrimination and auditory sensory behaviours in autism spectrum disorders. *Neuropsychologia,* *47,* 2850–2858. 10.1016/j.neuropsychologia.2009.06.015. 10.1016/j.neuropsychologia.2009.06.015 19545576

[CR35] Koolen S, Vissers CTW, Egger JIM, Verhoeven L (2014). Monitoring in language perception in high-functioning adults with autism spectrum disorder: Evidence from event-related potentials. Clinical Neurophysiology.

[CR36] Lepistö T, Nieminen-von Wendt T, von Wendt L, Näätänen R, Kujala T (2007). Auditory cortical change detection in adults with Asperger syndrome. Neuroscience Letters.

[CR37] Liikkanen LA (2012). Musical activities predispose to involuntary musical imagery. Psychology of Music.

[CR38] Lima, C. F., Lavan, N., Evans, S. Agnew, Z, Halpern, A. R., Shanmugalingam, P … & Scott, S. K. (2015). Feel the noise: Relating individual differences in auditory imagery to the structure and function of sensorimotor systems. *Cerebral Cortex, 25*, 4638–4650. 10.1093/cercor/bhv134. 10.1093/cercor/bhv134 PMC4816805 26092220

[CR39] Mayer JL, Hannent I, Heaton PF (2016). Mapping the developmental trajectory and correlates of enhanced pitch perception on speech processing in adults with ASD. Journal of Autism and Developmental Disorders.

[CR40] McCleery JP, Ceponiene R, Burner KM, Townsend J, Kinnear M, Schreibman L (2010). Neural correlates of verbal and nonverbal semantic integration in children with autism spectrum disorders. Journal of Child Psychology and Psychiatry and Allied Disciplines.

[CR41] McKelvie SJ (1995). The VVIQ as a psychometric test of individual differences in visual imagery vividness: A critical quantitative review and plea for direction. Journal of Mental Imagery.

[CR42] Miron O, Beam AL, Kohane IS (2018). Auditory brainstem response in infants and children with autism spectrum disorder: A meta-analysis of wave V. Autism Research.

[CR43] Mody M, Manoach DS, Guenther FH, Kenet T, Bruno KA, McDougle CJ, Stigler KA (2013). Speech and language in autism spectrum disorder: A view through the lens of behaviour and brain imaging. Neuropsychiatry.

[CR44] Molnar-Szakacs I, Heaton P (2012). Music: A unique window into the world of autism. Annals of the New York Academy of Sciences.

[CR45] Mottron L, Peretz I, Belleville S, Rouleau N (1999). Absolute pitch in autism: A case study. Neurocase.

[CR46] Ockelford A, McPherson GE (2015). The potential impact of autism on musical development. The child as musician: A handbook of musical development.

[CR47] Patel AD, Rebuschat P, Rohrmeier M, Hawkins J, Cross I (2012). Language, music, and the brain: A resource-sharing framework. Language and music as cognitive systems.

[CR48] Pfordresher PQ, Halpern AR (2013). Auditory imagery and the poor pitch singer. Psychonomic Bulletin & Review.

[CR49] Pijnacker J, Geurts B, van Lambalgen M, Buitelaar J, Hagoort P (2010). Exceptions and anomalies: An ERP study on context sensitivity in autism. Neuropsychologia.

[CR50] Pruitt TAS, Halpern AR, Pfrordressher PQ (2019). Covert singing in anticipatory auditory imagery. Psychophysiology.

[CR51] Roberts, T. P. L., Cannon, K. M., Tavabi, K., Blaskey, L., Khan, S. Y., Monroe, J. F., … & Edgar, J. C. (2011). Auditory magnetic mismatch field latency: A biomarker for language impairment in autism. *Biological Psychiatry*, *70*, 263–269. 10.1016/j.biopsych.2011.01.015. 10.1016/j.biopsych.2011.01.015 PMC3134608 21392733

[CR52] Rouder JN, Speckman PL, Sun D, Morey RD, Iverson G (2009). Bayesian t tests for accepting and rejecting the null hypothesis. Psychonomic Bulletin & Review.

[CR53] Schacter DL, Norman KA, Koutstaal W (1998). The cognitive neuroscience of constructive memory. Annual Review of Psychology.

[CR54] Schönbrodt FD, Perugini M (2013). At what sample size do correlations stabilize?. Journal of Research in Personality.

[CR55] Schwartz S, Shinn-Cunningham B, Tager-Flusberg H (2018). Meta-analysis and systematic review of the literature characterizing auditory mismatch negativity in individuals with autism. Neuroscience and Biobehavioral Reviews.

[CR56] Scott FJ, Baron-Cohen S (1996). Imagining real and unreal things: Evidence of a dissociation in autism. Journal of Cognitive Neuroscience.

[CR57] Scott SK, Rosen S, Beaman CP, Davis JP, Wise R (2009). The neural processing of masked speech: Evidence for different mechanisms in the left and right temporal lobes. Journal of the Acoustical Society of America.

[CR150] Sharda, M., Tuerk, C., Chowdhury, R., Jamey, K., Foster, N., Custo-Blanch, M., … & Hyde, K. (2018) Music improves social communication and auditory–motor connectivity in children with autism. *Translational Psychiatry,**8,* 231. 10.1038/s41398-018-0287-3. 10.1038/s41398-018-0287-3 PMC6199253 30352997

[CR58] Sota S, Hatada S, Honjyo K, Takatsuka T, Honer WG, Morinobu S, Sawada K (2018). Musical disability in children with autism spectrum disorder. Psychiatry Research.

[CR59] Stanutz S, Wapnick J, Burack JA (2014). Pitch discrimination and melodic memory in children with autism spectrum disorders. Autism.

[CR60] Surprenant AM, Neath I (2003). Principles of memory.

[CR61] Tryfon A, Foster NE, Ouimet T, Doyle-Thomas K, Anagnostou E, Sharda M, Hyde KL (2017). Auditory-motor rhythm synchronization in children with autism spectrum disorder. Research in Autism Spectrum Disorders.

[CR62] van Doorn, J., van den Bergh, D., Bohm, U., Dablander, F., Derks, K., … & Wagenmakers, E. (2019). The JASP guidelines for conducting and reporting a Bayesian analysis. 10.31234/osf.io/yqxfr. 10.3758/s13423-020-01798-5 PMC8219590 33037582

[CR63] Whitehouse AJO, Bishop DVM (2008). Do children with autism ‘switch off’ to speech sounds? An investigation using event-related potentials. Developmental Science.

[CR64] Williamson VJ, Jilka SR (2014). Experiencing earworms: An interview study of involuntary musical imagery. Psychology of Music.

